# Supplementary material for: CFTR Modulation Reduces SARS-CoV-2 Infection in Human Bronchial Epithelial Cells
Source: Cells. 2022 Apr 15;11(8):1347. doi: 10.3390/cells11081347 (PMC9028056; doi:10.3390/cells11081347)
Supplement: Supplementary file 1 [file cells-11-01347-s001.zip › Supplementary Figure S1.pdf]

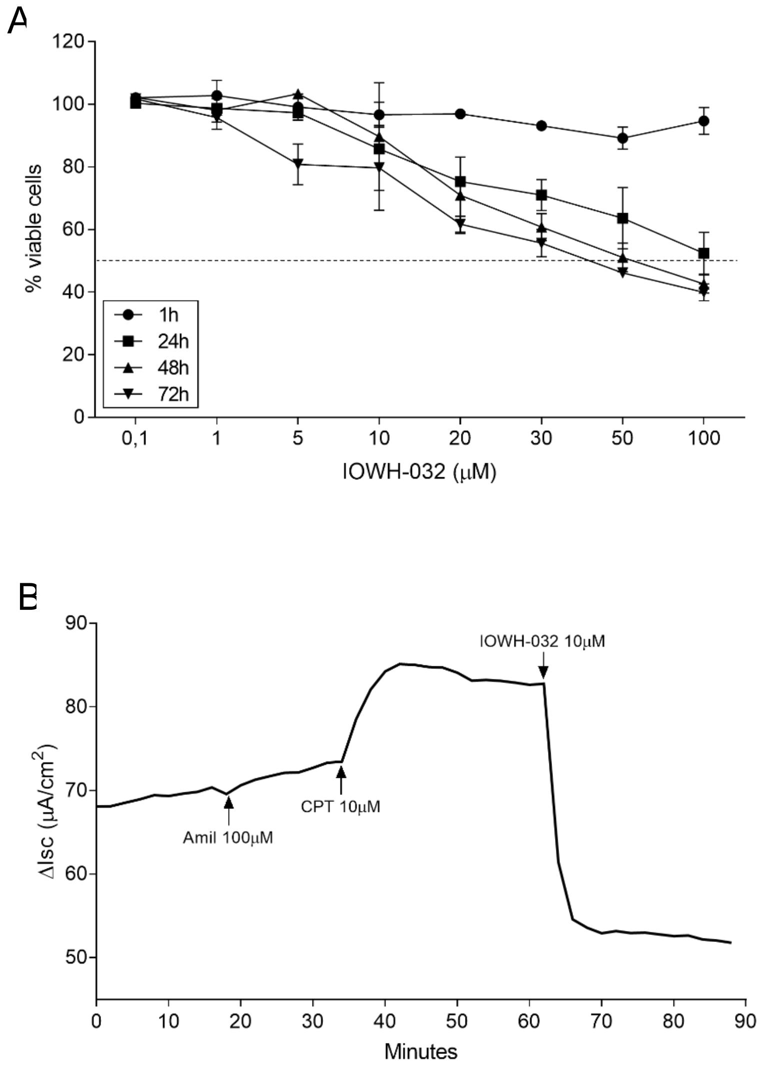

Supplementary Figure S1. Toxicity and effectiveness of CFTR inhibition by IOWH-032 in CFBE41o- WT cells. (a) To access IOWH-032 toxicity on live cells, we have measured the viability (%) of CFBE41o- treated with different concentration of the molecule analyzed at different time point by CellTiter 96® Aqueous One Solution Cell Proliferation Assay (Promega). The dashed line represents the LD50 (median lethal dose). ODs acquired at 495nm were normalized to DMSO alone and converted in percentage. Data are presented as mean ± SD from independent experiments (n=3). (b) Example of short-circuit current (Isc) measurement tracing used to prove the efficacy of IOWH-032 in inhibiting the CFTR channel (n=3). CFBE41o- cells were seeded on Costar Transwell® inserts (Corning, NY, USA) and grown in air-liquid interphase (ALI) for 2 weeks. Filters were mounted on a slider in an Ussing chamber and Isc was measured with a EVC4000 multi-channel voltage/current clamp (WPI, World Precision Instruments). Meyler saline buffer solution was used to fill the two half chambers. Subsequently, the ENaC blocker Amiloride (100 μM), the cAMP analog CPT (FSK; 10 μM) and the CFTR inhibitor IOWH-032 (10 μM) were added both to the apical and the basolateral sides. The tracings were recorded with PowerLab (8/35, AD Instruments) and data analysis were performed using Labchart v8 software (AD Instruments).
