# Supplementary material for: CFTR Modulation Reduces SARS-CoV-2 Infection in Human Bronchial Epithelial Cells
Source: Cells. 2022 Apr 15;11(8):1347. doi: 10.3390/cells11081347 (PMC9028056; doi:10.3390/cells11081347)
Supplement: Supplementary file 1 [file cells-11-01347-s001.zip › Supplementary Figure S2.pdf]

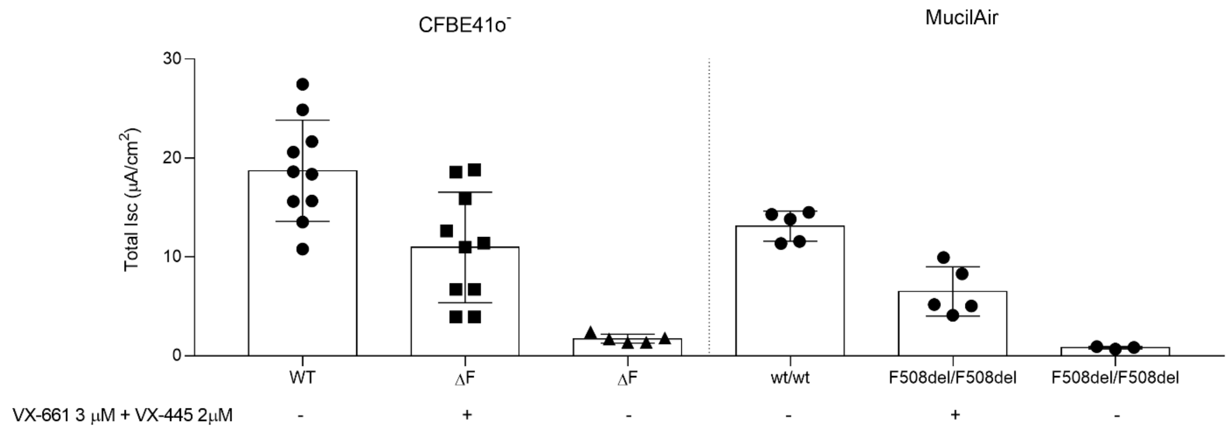

Supplementary Figure S2. CFTR function in CFBE41o- cells and in MucilAir™. Total Isc values (µA/cm^2) were calculated and plotted for both WT and ΔF CFBE41o-cells and wt/wt-CFTR and F508del/F508del-CFTR MucilAir with and without CFTR-modulators (VX-661+VX-445) correction. For CFBE41o- ΔF cells (n=10) the restored function after CFTR-correction was ~50% of CFBE41o- WT cells (n=10) as for the F508del/F508del-CFTR MucilAir™ (n=5) which rescue was ~45% of wt/wt-CFTR MucilAir™ (n=5).
