# Supplementary material for: CFTR Modulation Reduces SARS-CoV-2 Infection in Human Bronchial Epithelial Cells
Source: Cells. 2022 Apr 15;11(8):1347. doi: 10.3390/cells11081347 (PMC9028056; doi:10.3390/cells11081347)
Supplement: Supplementary file 1 [file cells-11-01347-s001.zip › Supplementary Figure S3.pdf]

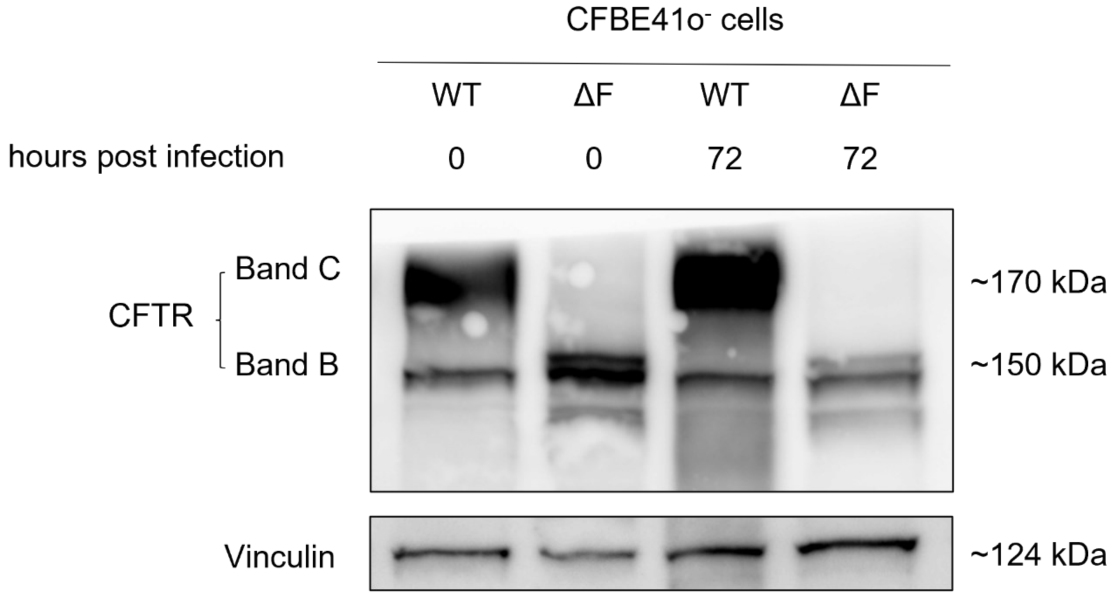

Supplementary Figure S3. Protein expression levels of CFTR in WT and ΔF CFBE41o- cells before and after SARS-CoV-2 infection. Total protein lysates were solubilized in 4x Laemmli sample buffer (4% SDS, 5% β-mercaptoethanol, 20% glycerol, 0.0025% bromophenol blue, 0.16 M Tris-HCl, and pH 6.8) with 125 mM DTT and incubated at 37 °C for 15 minutes. Then, protein samples were analyzed by SDS-PAGE, transferred to a PVDF membrane (Bio-Rad) and probed with α-CFTR monoclonal antibodies (mAb) 450, 570, and 596 (CFTR Antibody Distribution Program, Cystic Fibrosis Foundation, UNC-Chapel Hill) (1:1000 dilution each) overnight at 4°C. CFBE 41o- WT cells show the presence of both C (glycosylated) and B (unglycosylated) CFTR bands while in CFBE 41o- ΔF cells only the B band is evaluable. Vinculin mouse monoclonal antibody was used as a loading control.
